# Body size variation of the ant *Lasius niger* along a metal pollution gradient

**DOI:** 10.1007/s11356-019-04811-0

**Published:** 2019-05-07

**Authors:** Irena M. Grześ, Mateusz Okrutniak, Monika Gorzałczany, Piotr Piszczek

**Affiliations:** 1 Department of Environmental Zoology, Institute of Animal Science, University of Agriculture in Krakow, al. Mickiewicza 24/28, 30-059 Kraków, Poland; 2 Institute of Botany, Faculty of Biology and Earth Sciences, Jagiellonian University, Kopernika 27, 31-501 Kraków, Poland

**Keywords:** Ants, Trace metals, Body size, Pollution, Adaptation, Colony personality

## Abstract

The phenotypic diversity of ant workers plays a fundamental role in their biology. In this study, we asked if the body size variation of monomorphic workers of the ant *Lasius niger* (Formicidae) responds adaptively to metal pollution in a post-mining metal-polluted area. Nest samples of workers were collected along a pollution gradient to calculate the within-colony variance in body size (expressed as maximum head width, HW). The results showed that the body size variation of *L. niger* was unrelated to the pollution index but demonstrated considerable variation between colonies even within the same study site. We suggest that the differences in morphological diversity between the colonies of *L. niger* could be shaped by colony personality traits, i.e., by colony-specific foraging and/or the feeding efficiency of nursing workers. The study supports previous findings, showing that morphological traits in *Lasius* ants are weakly related to environmental metal pollution.

## Introduction

The morphological diversity of ant workers is believed to enhance colony performance. The association between the morphological variation of workers and colony fitness was confirmed mainly for ants that maintain morphologically distinguishable worker subcastes (Jaffe et al. 2007; Evison and Hughes 2011). Nevertheless, the vast majority of ants, even the most successful species, have monomorphic workers of limited size variation (Oster and Wilson 1978). Testing the adaptive meaning of body size variation in such species as well as its plasticity in response to environmental stress showed contradictory results (Beshers and Traniello 1994; Billick and Carter 2007; Modlmeier and Foitzik 2011; Colin et al. 2017). Some authors suggested that we need to improve our understanding of body size diversity among ants, by including research at the species and colony level (Gouws et al. 2011; Wills et al. 2018).

Worker body size and its distribution can be influenced by several potentially interacting factors acting within or outside the colony (reviewed in Wills et al. 2018). Genetic background, hormonal regulation, temperature, photoperiod, and predation pressure as well as competition with other ants are among the most important body size determinants of ants (Davidson 1978; Herbers 1980; Wheeler and Nijhout 1981; Bargum et al. 2004; Kipyatkov et al. 2004; Yang et al. 2004; Linksvayer 2006; Abril et al. 2010). For example, environment rich in food resources positively affects the body size of ant workers (Deslippe and Savolainen 1995). Additionally in ants, social organization also greatly contributes to the size variation. Typically, polymorphic ants produce small workers during early stages of colony founding, while the production of large workers grows as colony age increases (Hölldobler and Wilson 1990).

Ants are a common component of the fauna in metal polluted areas (Eeva et al. 2004; Grześ 2009; Belskaya et al. 2017; Frizzi et al. 2017). Body size variation in ants in such areas may be associated with metal pollution level for several reasons. First, pollution may influence the body size of ants, which relates to selection mechanisms leading to optimal ant size under such conditions. If natural selection acts on body size, its diversity should decrease proportionally to an increase in the local contamination level. Morphological diversity can be also affected indirectly, because the ants may be adaptively responding to reduced food resources; high diversity of workers could allow for more efficient foraging, enhancing colony fitness. Alternatively, pollution level and body size diversity may not be significantly correlated. It is likely that variation in body size is strongly colony-dependent due to the colony personality traits (sensu Wilson et al. 1994; Gosling 2001). It has been proven that ant colonies can differ considerably from one another in a consistent manner even within the same population in terms of their efficiency in foraging, brood feeding, or thermal regulation (DiRienzo and Dornhaus 2017; Pinter-Wollman 2012). Between-colony variation in body size diversity can be also driven by the genetic background of a colony. Polygyny and polyandry should significantly increase the size diversity of workers. One could also hypothesize that the body size variation of ants relates to the size of potential prey items. In consequence, the body size variation of workers may be better explained by the local diversity of prey around the nest than by the pollution level of the site. The high between-colony differences caused by the factors mentioned above may mask the positive or negative effect of metals on the body size diversity of workers.

In 2015, we published a study on the common garden ant *Lasius niger* (Grześ et al. 2015a) that addressed the within-nest distribution of body size in the same polluted area as that used in the present research. Apart from the skewness of the within-nest distribution curves, all other statistics, including the variation coefficient, did not significantly relate to the pollution level. In that study, we considered only 19 replicates; therefore, the results which were not significant were rather conservative and would constitute a type-II error. On the other hand, our most recent and on-going studies of ants originating from non-polluted areas showed that the trends in body size may be weak but still significant and likely of biological importance (Grześ et al. 2016). However, these results were detected when a big dataset was considered.

This study concerns morphological diversity of common garden ant *Lasius niger* along a metal-pollution gradient in a post-mining area, using a relatively high number of replicates, giving the chance to confirm the association between pollution and variation, if it truly exists. The study area is contaminated by high concentrations of zinc, cadmium, and lead. *L. niger* is very common in the investigated area. We expressed morphological diversity as the body size variations of the workers within the nests. We quantified their size by measuring head width, a standard measure of size in ants. We ask the question: “how flexible is body size diversity as a response to metal pollution”?

## Materials and methods

### Study area

**Fig. 1:**
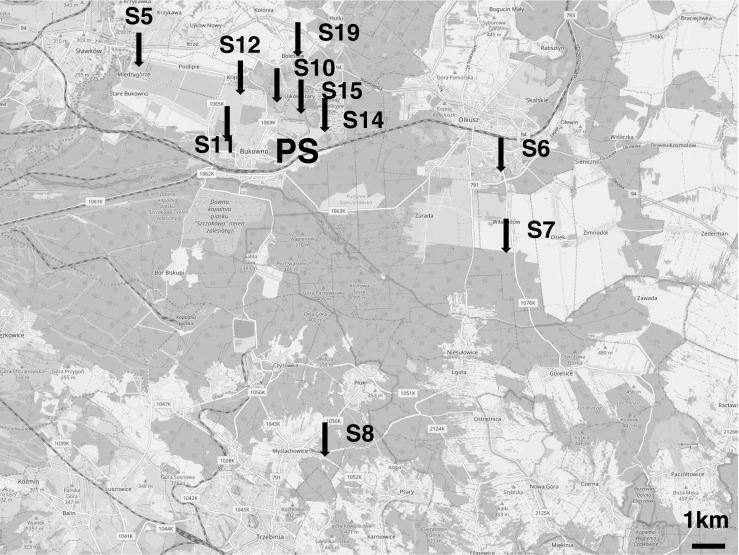
Study area showing the location of ten sampling sites in the post-mining area of the Bolesław zinc-and-lead smelter (© authors OpenStreetMap). Abbreviation refers to the pollution source (PS)

The study sites were located in the post-mining area of the Bolesław zinc-and-lead smelter in southern Poland. In a preliminary study, 10 study sites were established along the Zn and Cd soil pollution gradient covering formerly cultivated fields (eight sites) and industrial wastelands (two sites). The gradient extended from 0.7 to 10 km from the pollution source (Fig. 1). Most sites are currently covered by mixed ruderal-meadow-grasslands vegetation and have a xerothermic character with diversified types of plant communities, of which the *Arrhenatheretum elatioris* association was most frequent. Brown loamy or poorly loamy, neutral to base-rich soils, dominated the sites (Woch 2011). The total metal concentrations in the topsoil at the sites used in this study ranged from 158.36 to 16,984.90 mg/kg d.w. for Zn and from 3.36 to 63.86 mg/kg d.w. for Cd (Grześ et al. 2015a). Because ants in natural conditions are exposed to metal pollution via the trophic route (Maavara et al. 1994), we expressed the pollution index of each of the 10 study sites as the total concentration of Zn in a random sample of field-collected invertebrates that could potentially comprise the diet of *L. niger*. The transfer of metals from soil to invertebrates is well documented (Graff et al. 1997; Wilczek et al. 2005; van Straalen et al. 2005; Butovsky 2011; Ardestani and van Gestel 2013; Boshoff et al. 2013; Ding et al. 2013). The total concentration of Zn in invertebrates was used as a convenient pollution index in our previous studies performed in the Bolesław smelter area. The concentrations of both contaminants in invertebrates and in the soil were correlated (Grześ et al. 2015a; Grześ and Okrutniak 2016). The lowest and the highest Zn concentrations detected in the invertebrates were 134.86 and 1545.11 mg/kg d.w., respectively. Detailed metal concentrations in the soil and invertebrates, as well as a botanical description of all sites, are reported in Grześ et al. (2015a; see sites S5-S8, S10-S12, S14, S15 and S19).

### Sampling

*Lasius niger* (L. 1758) is the dominant ant species inhabiting the investigated area; its relative abundance accounted for more than 70% of ants (Grześ 2009). This strictly monodomous species builds mineral nest mounds and is both carnivorous and aphidicolous. Nuptial flights take place from July to late August (Czechowski et al. 2012). Samples of *L. niger* workers were collected from mature colonies in the end of July 2016. In each of the 10 sites, 10 colonies were standardized for relatively large mound diameter for statistical purposes. The mound diameter ranged between 40 and 60 cm. In total, 100 colonies were investigated. A random sample of about 30 workers was collected using a plastic exhaustor just beyond the opening of the nest until the first sexuals were observed. The samples were stored at − 5 °C until measured.

### Morphological measurements

The body size of ants was expressed as the maximum head width across the eyes (HW sensu Czechowski et al. 2012). Head width is commonly used as a convenient index of body size in a number ant species of the *Formicinae* subfamily (Yamauchi et al. 2001; Fjerdingstad 2005; Schwander et al. 2005; Aron et al. 2009). In total, 3000 workers were measured. Measurements were performed under a metallographic microscope Met-153 (Motic, China, × 100 magnification) with a digital camera (Huvitz, Korea). The head width of each ant was measured based on a digital photograph to the nearest 0.0001 mm using Panasis, ver. 2.4.2, Huvitz.

### Statistical procedures

Two independent analyses were performed.

1. We tested the relationship between pollution and body size diversity of *L. niger* using generalized regression model. We expressed body size diversity as intra-colony variances in head width (HW). Thus, we obtained one measure for each colony. Then, we regressed the calculated variances against metal pollution index, i.e., the total concentration of Zn in a random sample of field-collected invertebrates that could potentially comprise the diet of *L. niger*. The distribution of the calculated variances was found to be right-skewed; therefore, we used generalized regression model (GLMs) with gamma distributed dependent variable and identity link function (Welder 1972). Model was fitted using the STATISTICA program (StatSoft Inc., 2001).

As ants are social insects living in colonies, we assumed that each colony should be treated as an independent replicate. Because 10 colonies were chosen at each site, which results in a special dependence of colonies, the colony may seem a pseudoreplication for each study site. To avoid this problem, we followed the advice of Davies and Gray (2015) by limiting our conclusions to the specific gradient at a specific site and not to the metal pollution per se. Therefore, we are aware that our final findings are rather more of a hypothesis for future studies than irrefutable evidence.

2. In order to analyze which factor—the site of origin or the colony from which ants were sampled—contributes the most variance in individual body size (head width), the variance components analysis was performed. Regarding the hierarchical nature of the data, the factor “colony” nested in the random factor “site” was incorporated in the model as a categorical random factor. The output of the analysis is the percentage of the total variance represented by each factor. The analysis was validated by testing whether the residuals met the assumptions of normality using the Shapiro-Wilk’s test using Statgraphics Centurion XV.

## Results

**Fig. 2:**
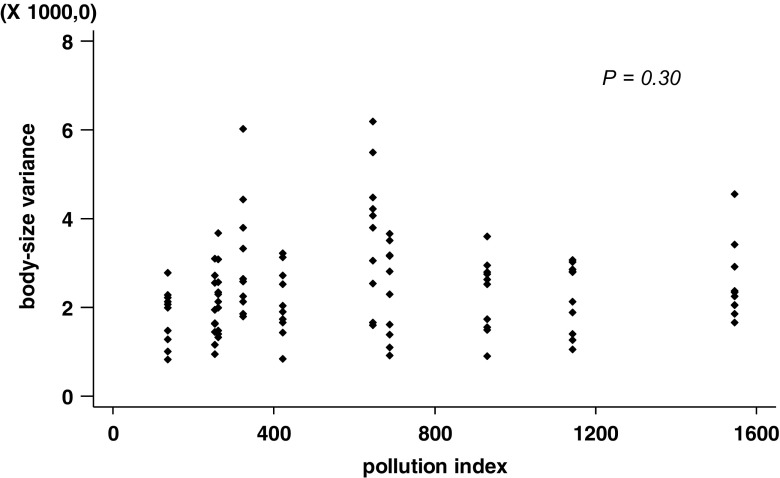
The relationship between the variance in the size of head width in different colonies and the pollution index of the site. The pollution index is expressed as the total Zn concentration (mg/kg d.w.) in a random sample of small invertebrates collected at each of 10 study sites. Each point represents the variance of one colony (*N* = 100)

**Fig. 3:**
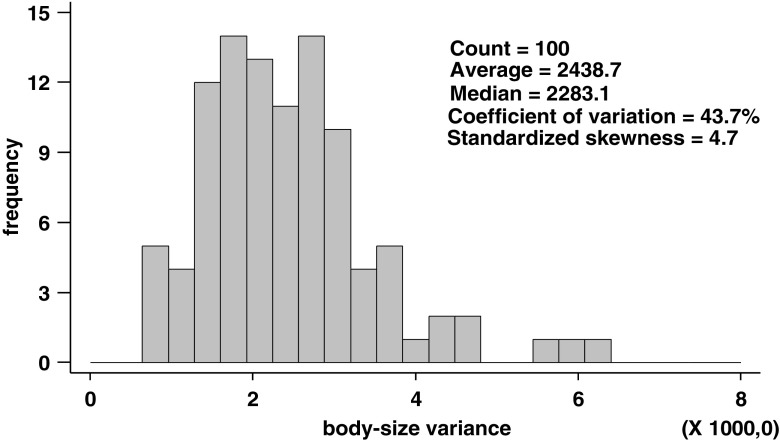
The histogram of within-colony variances in the body size and the descriptive statistics of the distribution

No significant correlation was found between the pollution index and intra-colony variance in body size (*N* = 100, *χ*^2^ = 1.06, *P* = 0.30, Fig. 2), indicating that metal pollution does do not diminish nor increase the body size variation of the investigated ant. The distribution of variances is right-skewed (standardized skewness = 4.7, Fig. 3), indicating that ants of several nests are highly variable irrespective of the metal pollution index of their origin. The contribution of the factors colony and site represents 20.8% and 6.8% of the total variation in body size, respectively. The 72.4% of the total variance is unexplained by the above factors (error factor).

## Discussion

The study was done to assess the body size diversity in the common garden ant at 10 independent sites forming a well-known metal pollution gradient. The body size variance calculated for each of the 100 nests turned out to be not associated with the pollution index of the site of origin, suggesting a relatively low flexibility of body size variation in the response to environmental metal pollution. We indicated that more variance was attributable to the differences between the colonies than to the differences between the sites with different pollution values. Because body size diversity did not increase or decrease with increasing pollution, it seems that *L. niger* workers do not adapt to metal pollution by extending or narrowing body size range. We also found no support for the hypothesis that metal pollution selects the ants for the particular body size. Although the toxic effects of trace metal pollution on ants are recognized (e.g., Sorvari et al. 2007 for disturbance of ant immune response system), evidence for the adaptation to environmental metal pollution in an evolutionary sense, or at least for increased metal tolerance, is very limited (Grześ 2010; Grześ and Okrutniak 2016). Proof of increased Zn tolerance has been demonstrated for the adult workers of *Myrmica rubra*, showing that workers collected in polluted sites were less metal sensitive than those collected in control areas (Grześ 2010).

The within-colony body size variances do not correlate with the pollution index, but the values of these variances are diversified between the colonies. This is consistent with the variance components analysis showing that a greater amount of variance in body size is due to the differences between the colonies than to the differences between the sites of origin. This means that colony-dependent factors, presumably due to genetic background or colony personality traits, are more important in shaping variation in body size of the investigated species than site-specific external environmental conditions. These factors contribute to the between-colony differences in body size variation so that the effect of pollution is not statistically detectable although we used relatively big data set. Genetic diversity at the colony level of many ant species increases body size diversity (Fjerdingstad and Crozier 2006). The investigated species has one reproductive female in the nest, but the number of its sexual partners may differ between nests, providing a variable number of parental lines of the offspring (Fjerdingstad and Keller 2004). This may at least partially explain the genetic basis for the observed between-colony differences in body size diversity. We suggest that observed intra-colony variance in the body size could be treated as a secondary effect of colony personality.

It cannot be excluded that other non-social environmental factors unrelated to pollution but acting locally could also contribute to the observed high variation in the body size between the colonies. Assuming that the study sites are diversified in the respect of microhabitats, the colonies may be subjected to different temperature and humidity. It was found the nest temperature effects of worker size in *Formica aquilonia* (Sorvari and Hakkarainen 2009). It is also possible that the colony-specific body size diversity is driven by the adjustment of worker size and the size of potential preys around the nest. However, testing this hypothesis would require detailed data on local food variation and their contribution to the diet of ants of each colony.

The presented results are in agreement with those obtained in our previous studies on *Lasius* ants indicating that their morphological characteristics are usually unrelated to the pollution level. Based on correlative studies, we found no association between average body size in mature or immature colonies of *L. niger* (Grześ et al. 2015a, 2015b). Similarly, the asymmetry of the eyes of *Lasius flavus* was not associated with pollution, suggesting the ability of ants to maintain a stable and repetitive trajectory of eye development during the larval stage (Grześ et al. 2015c). Comparing the studies of other authors, ants seem to be much less sensitive to pollution-induced morphological changes than carabids in metal-polluted sites (Maryański et al. 2002; Łagisz 2008) as well as in urbanized areas (Weller and Ganzhorn 2004; Sukhodolskaya 2013). Body size decrease in carabids might be explained by the limited resource availability during larval stage development or by the energy reallocation from growth to detoxification (Łagisz 2008). The environmentally induced changes in morphological parameters were postulated to be permissible indicators in metal pollution risk assessment (Skaldina and Sorvari 2017). However, as the morphological diversity of *L. niger* workers was not associated with the pollution index at the site of their origin, the present study gives no good reasons for recommending size variation of this species as an efficient metal bioindicator.

To summarize, variation in the body size of the monomorphic ant *Lasius niger* was unrelated to the pollution index along the investigated pollution gradient, but demonstrated considerable variation between colonies even within the same study site. It seems that colony-dependent factors are more important in shaping variation in the body size of the investigated species than the pollution level. The study supports previous findings showing that morphological traits in ants are weakly related to environmental metal pollution.
